# Anisotropic epitaxial ZnO/CdO core/shell heterostructure nanorods

**DOI:** 10.1186/1556-276X-7-626

**Published:** 2012-11-14

**Authors:** Sang Hyun Lee, Chad M Parish, Jun Xu

**Affiliations:** 1 Oak Ridge National Laboratory, P.O. Box 2008, Oak Ridge, TN 37831, USA

**Keywords:** ZnO nanorod, CdO, Heterojunction, Anisotropy

## Abstract

Various surface structures and polarities of one-dimensional nanostructures offer additional control in synthesizing heterostructures suitable for optoelectronic and electronic applications. In this work, we report synthesis and characterization of ZnO-CdO nanorod-based heterostructures grown on a-plane sapphire. The heterojunction formed on the sidewall surface of the nanorod shows that wurtzite ZnO {1010} planes are interfaced with rocksalt CdO {100}. This is evidently different from the heterojunction formed on the nanorod top surface, where a ZnO (0001) top plane is interfaced with a CdO (111) plane. Such anisotropic heterostructures are determined by different surface structures of the nanorods and their polarities. Revelation of such anisotropic heterojunctions will provide a clue for understanding charge transport properties in electronic and optoelectronic nanodevices.

## Background

Semiconductor heterojunctions serve as important gateways for charge transport in many optoelectronic devices, such as light-emitting diodes, photovoltaic solar cells, radiation/UV sensors, and electronic devices [1,2]. The heterojunctions formed by one-dimensional nanostructures are especially interesting because of the intrinsic large surface area and multiple crystal planes associated with these nanostructures [3-9]. For nanorods with large diameters ranging from 10 to 1,000 nm, quantum confinement hardly has appreciable effects, and their physical properties are determined, to a large extent, by their surfaces and the interfaces. Of critical importance for the formation of nanoscale heterojunctions are surface polarity and structural facets. Surface polarities of hexagonal group II-VI and III-V semiconductors, including AlN, InN, GaN, and ZnO, have been shown to associate with their anisotropic crystal properties [10,11]. These polarities affect both nanorod synthesis and electronic structure at the junction interface. For example, the Zn-polar faces determined the growth direction of ZnO nanorods along their c-directions [12]. Similarly, the multiple surface facets of a ZnO nanorod allow better matching of lattices with other semiconductors, leading to reduction of interfacial trap centers and resulting in enhancement of nanodevice performance. The combined effects of both polarity and structure are expected to produce favorable band energy profile for charge transport, as previously shown for GaN [13]. In this work, we intend to understand the formation of such an anisotropic heterojunction on wurtzite ZnO nanorod surfaces surrounded with rocksalt CdO.

For a hexagonal wurtzite ZnO nanorod, heterojunctions formed at the top and sidewall surfaces of the nanorod are expected to be obviously distinct because their crystal planes are different. Typically, the top surface of the ZnO nanorod is characterized by the (0001) structure with Zn polar. However, for the sidewall hexagonal surfaces of the nanorod, the crystal planes are expected to be nonpolar facets, such as {1010} or {1120}, due to relatively low surface energy [14]. Consequently, the heterojunctions formed with rocksalt-structured CdO on these surfaces are expected to be dramatically different, which would further affect the band energy profiles crossing the heterojunctions and enable the control of the related charge transport. In this work, ZnO/CdO nanorod heterostructures were synthesized and characterized on both the top and side surfaces of ZnO nanorods. The formation origin of the heterojunctions and their effects on charge transport are discussed. CdO was chosen as the outer shell because of the following reasons. First, CdO can serve as an n-type window or light absorption layer on transparent conducting oxide, such as Al- or Ga-doped ZnO, for solar cells [15,16]. Second, the energy band gap (2.3 eV at room temperature) of CdO can be tuned by ZnCdO formation under rationally controlled Zn/Cd ratio [17], which makes it a good candidate for a visible light emitters or lasers.

## Methods

Vertically aligned ZnO nanorods were grown on an a-plane sapphire substrate using catalytic growth in a horizontal quartz tube furnace. A thin Au film with 1 nm thickness as a catalyst was deposited on the substrate before the growth of ZnO nanorods using an e-beam evaporator. The substrate was located on a source boat containing 1:1 mixture of ZnO and graphite powders; the latter was employed for carbothermal reduction. During the growth, the source and substrate were maintained at a temperature of 900°C and under a constant flow of 200 sccm mixed gas of 10% pure oxygen in Ar at a pressure of 4 Torr. The as-grown ZnO nanorods have a typical diameter of about 150 nm and a length ranging from 10 to 15 μm. After replacing with another quartz tube, the nanorod sample was placed in the downstream of the Cd metal source at a distance of 3 cm. A CdO layer was deposited on the ZnO nanorods by evaporating the Cd metal at 450°C under the same conditions used for the ZnO nanorod growth unless specified otherwise.

## Results and discussion

**Figure 1:**
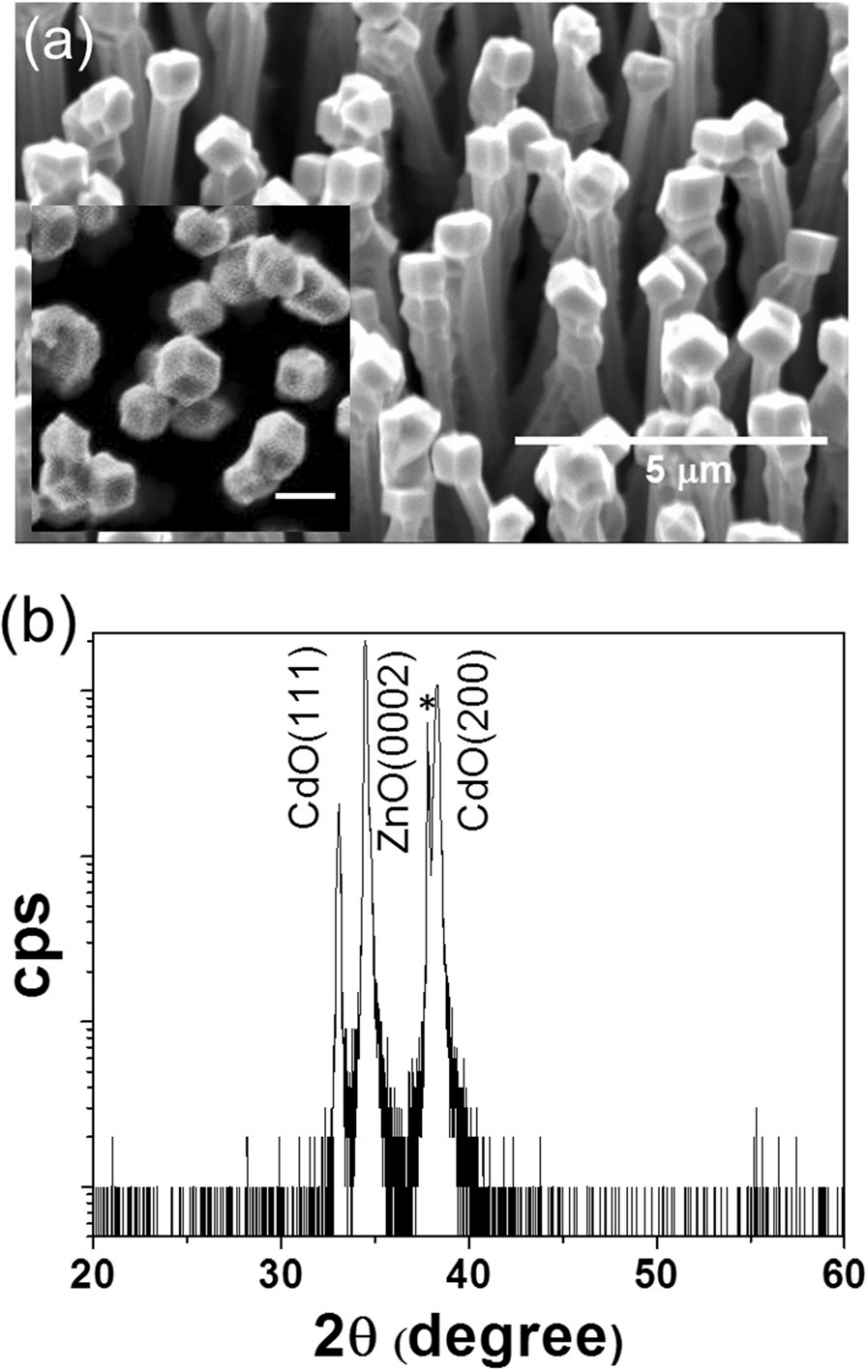
**SEM and XRD result of the ZnO/CdO core/shell nanorods grown on a Si substrate.** (**a**) SEM image and (**b**) XRD pattern. The inset in (**a**) shows the top-view image with a scale bar of 1 μm. The peak labeled with asterisk is ascribed to the Al_2_O_3_ (1120) diffraction plane (JCPDS Cards no.: 43–1484).

Figure 1a shows typical scanning electron microscope (SEM, Hitachi S4700, Hitachi, Ltd., Tokyo, Japan) images of the samples after CdO deposition on ZnO nanorods. Well-developed cubes with submicron size are clearly seen on the top of nanorods, which are visible from both side and top views (see the inset of Figure 1a). These submicron cubes represent equal aggregation of cubic rocksalt CdO cells. The diameter of ZnO nanorods after CdO deposition increases from 150 to about 300 nm. On closer visualization, we further noticed that the cubes exhibit specific angle with respect to the axial direction of ZnO nanorod, which is approximately 45°. X-ray diffraction (XRD, PANalytical X-ray diffractometer (Panalytical B.V., Almelo, The Netherlands)) spectrum of the ZnO/CdO sample, shown in Figure 1b, indicates the existence of rocksalt CdO on hexagonal ZnO (JCPDS Card nos.: 05–0640 and 36–1451). For ZnO, the spectrum exhibits only one peak at 34.4°, which is associated with the ZnO (0002) as a result of the growth of the ZnO nanorods along the vertical orientation on the a-plane sapphire substrate. In contrast, two peaks were observed for CdO, which are related to the CdO (111) and (100), respectively. These peaks indicate that there are two CdO crystal planes parallel to the ZnO (0001) plane.

**Figure 2:**
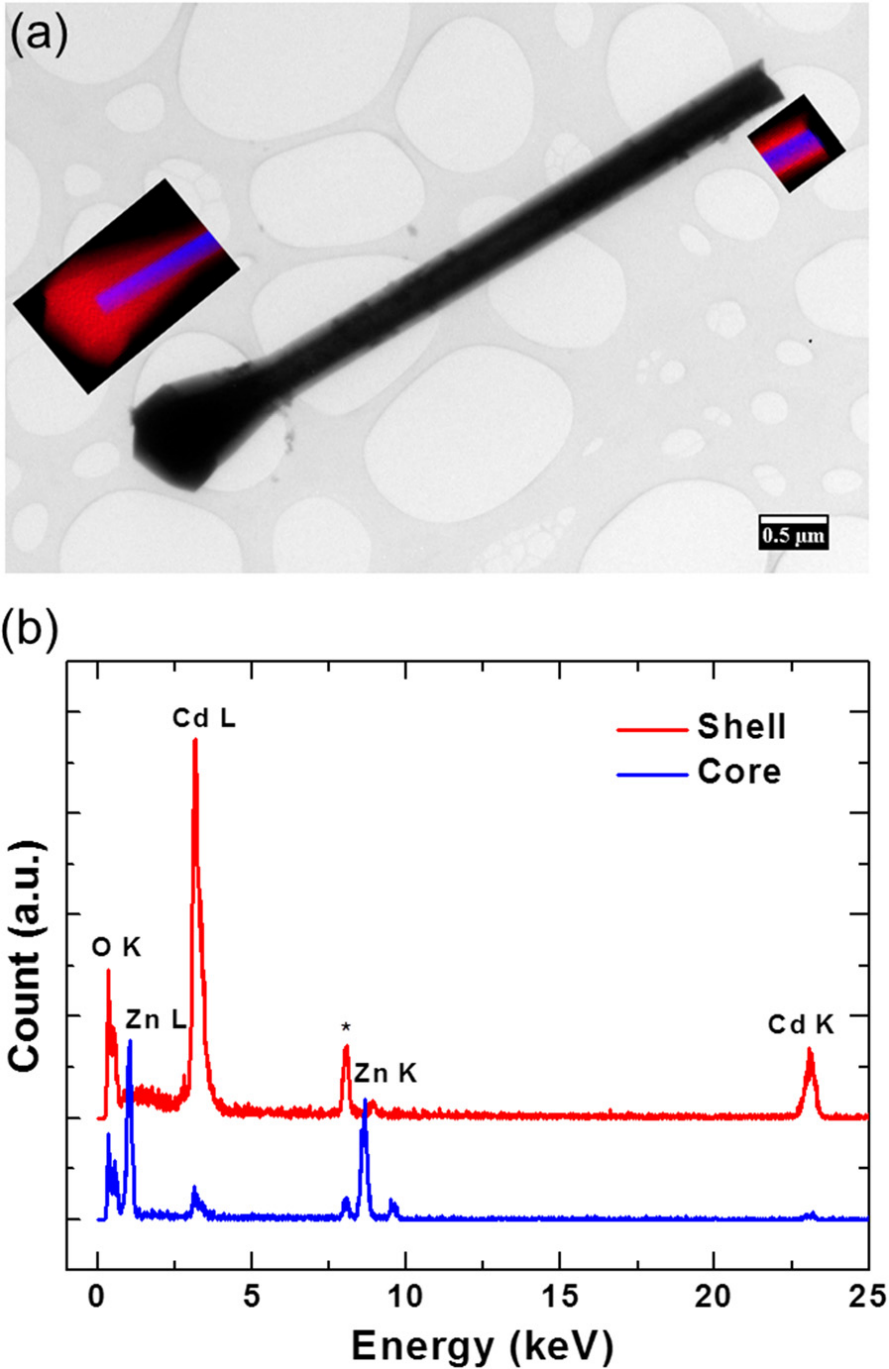
**TEM bright field image of the ZnO/CdO nanorod and the corresponding X-ray mapping.** Cd Kα (red) and Zn Kα (blue) clearly show the formation of core/shell heterostructure. The peak labeled with asterisk is CuK peak originating from the TEM grid.

Figure 2a shows a representative low-magnification transmission electron microscope (TEM) image of a single nanorod obtained by dispersing onto a lacy carbon film copper grid and imaging with a Philips CM200 TEM operated at 200 kV (Philips Electronics N.V., The Netherlands). The diameter of the core/shell nanorods gradually decreases from about 800 to 400 nm for the 1 to 2-μm portion from the tip of the nanorod. In addition to the cubic particles, the nanorod also exhibits a homogeneous CdO shell with a thickness of 130 nm surrounding the ZnO core. The chemical composition of the core and shell regions can be identified from energy dispersive X-ray (EDS) spectra shown in Figure 2b. Zinc and oxygen are dominant in the core region, whereas the shell is composed of cadmium and oxygen. Formation of core/shell structure is clearly evident from the elemental maps for both cadmium Kα (red) and zinc Kα (blue) X-rays at the top and the bottom of the nanorod inset to the TEM image shown in Figure 2a.

**Figure 3:**
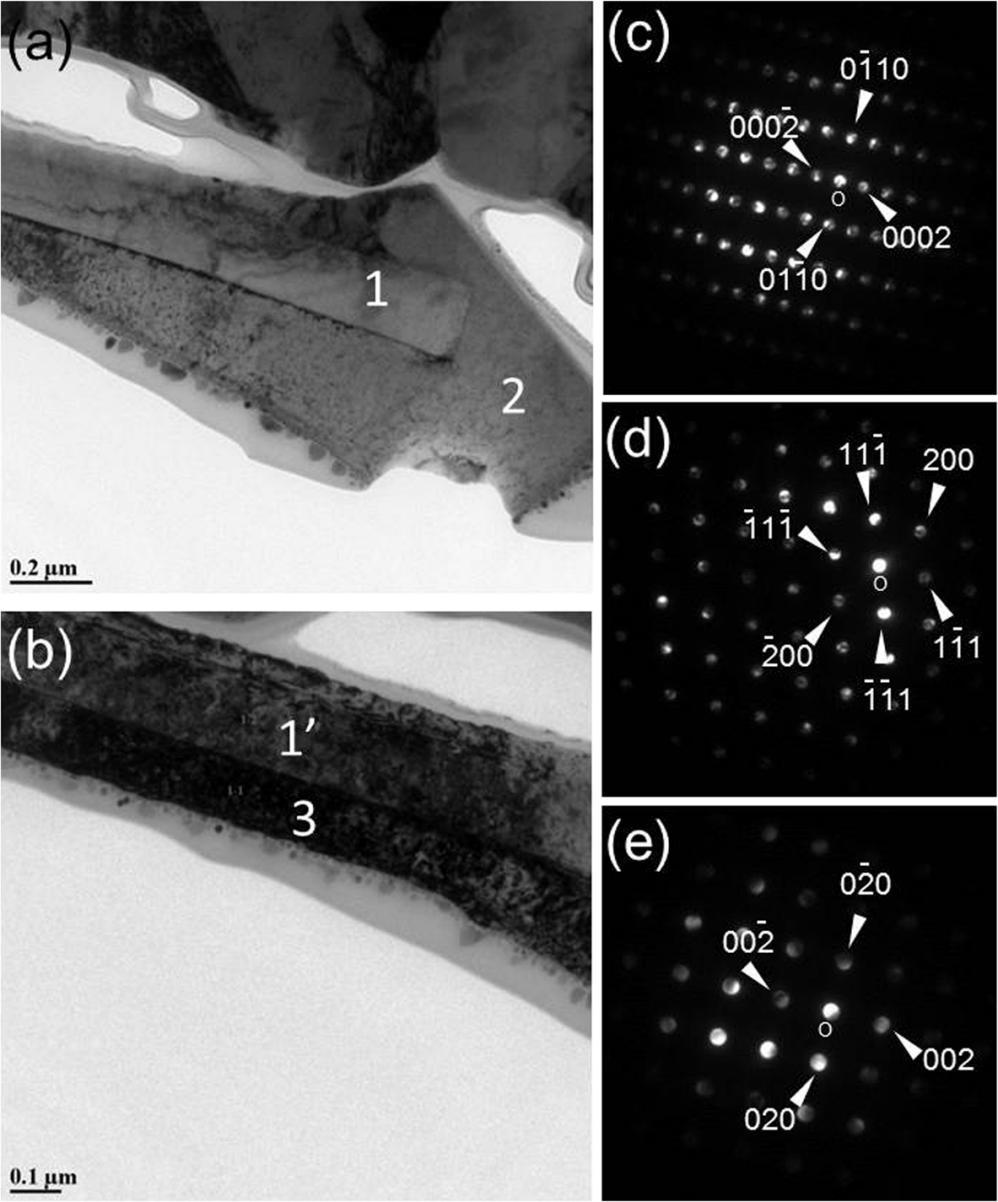
**TEM bright field image of the FIB thinned cross-section of ZnO/CdO nanorod.** (**a**) At the top and (**b**) the body as well as (**c**, **d**, and **e**) nanodiffraction patterns at the local area labeled 1 (1^′^), 2, and 3, which correspond to the ZnO core, CdO shells at tip, and at the side of ZnO nanorod, respectively.

Epitaxial relationship between the core and the shell structure was investigated using thinned nanorods with a Hitachi NB5000 focused ion beam-scanning electron microscopy tool. Specimens were milled to electron transparency with 40 keV Ga^+^ ions and then finally polished with 5 keV Ga^+^. Figure 3a,b shows the TEM images obtained from the tip and body part of the ZnO/CdO core/shell structure. Boundaries between the core and shell structure can be clearly seen in both images. The asymmetry of the shell and the core structures in the TEM images might be due to damage during the focused-ion beam (FIB) milling. Nanodiffraction analysis from the core, top, and side of the shell, which are labeled with 1, 2, and 3 in the TEM images, are shown in Figure 3c,d,e, respectively. The nanodiffraction patterns reveal that the ZnO nanorod only grows along the [0002] direction, consistent with the XRD measurement. On the other hand, different growth directions at the top and side of the nanorods were found for CdO: [111] on the top and {001} on the sidewall of ZnO. These TEM results confirm that two XRD CdO peaks are indeed associated with two different heterostructures (Figure1b).

**Figure 4:**
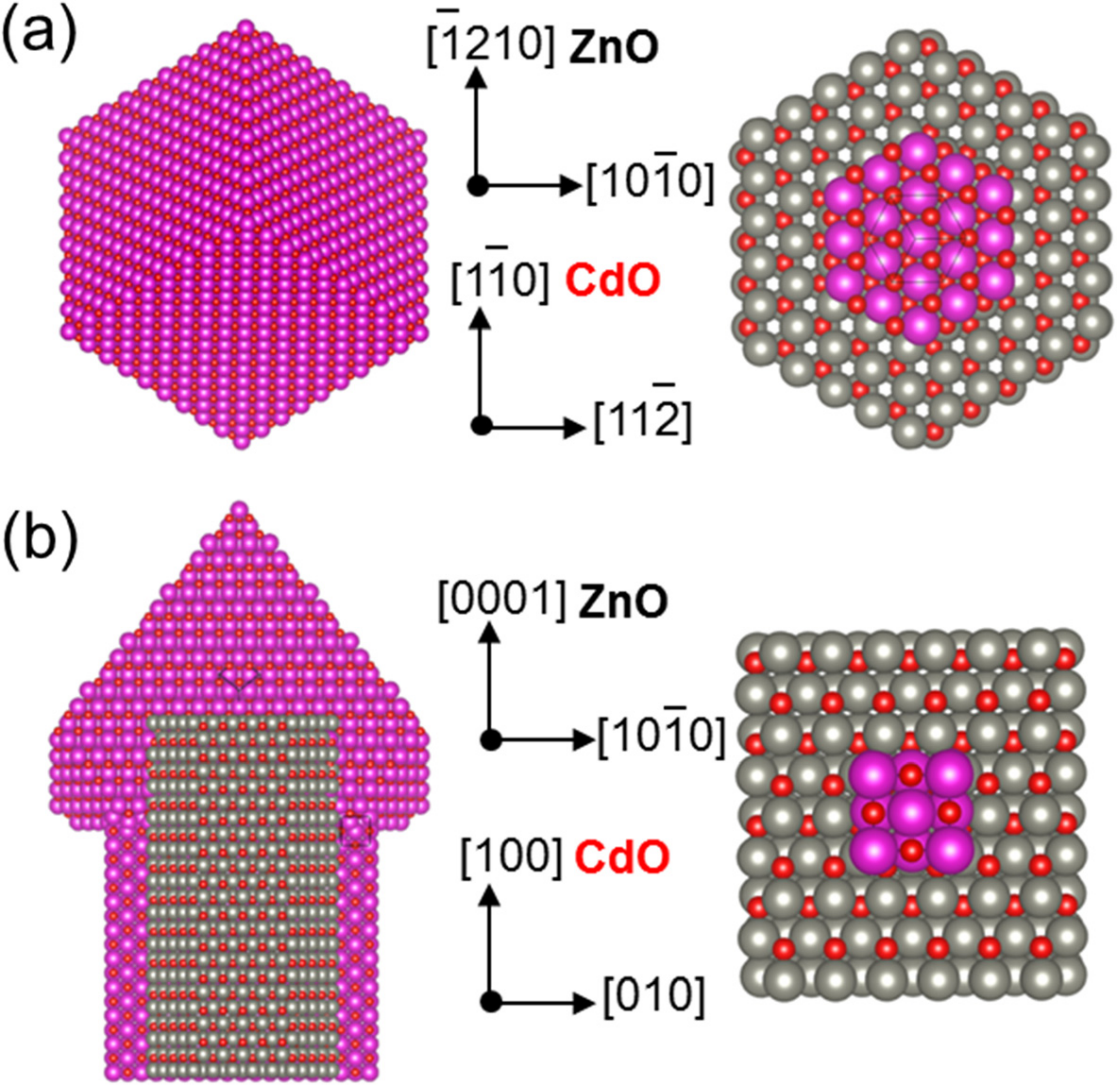
**Illustrations of atomic configurations and epitaxial orientations of CdO/ZnO.** (**a**) On the top and (**b**) side plane of ZnO nanorods. Gray, red, and purple sphere denotes zinc, oxygen, and cadmium atoms, respectively.

Figure 4 illustrates the schematic atomic configurations of CdO on ZnO top (001) and side (100) planes. Projected cubic CdO structure along the [111] growth direction exhibits a hexagonal shape on the ZnO (0001) plane, which is consistent with SEM image shown in Figure 1a. Consequently, epitaxial alignments of the heterostructure along the axial and radial directions are expected to be (111) CdO/(0001) ZnO, (311) CdO//(1010) ZnO, (001) CdO//(0001) ZnO, and (010) CdO//(1010) ZnO as shown in Figure 4. The epitaxial relationship on the polar and nonpolar surface of ZnO can explain favorable growth behaviors to minimize a lattice mismatch between CdO and ZnO according to the crystal plane. The lattice mismatches of both epitaxial interfaces are calculated to be about 1.8% and 10.9% (28), respectively. In addition, the origin of a thicker CdO layer on the top of ZnO than a shell layer on sidewall might be due to differences in the surface energies: higher for the polar surface of ZnO (0001) and CdO (111) and lower for the nonpolar surface of ZnO {1010} and CdO (100) [14,18]. Under thermodynamic equilibrium conditions, the crystal growth rate typically accelerates along the direction of crystal facets with a high surface energy, which occupy small areas in nanostructures [19].

## Conclusions

ZnO/CdO core/shell nanorods were synthesized using thermal vapor transport deposition and are characterized with XRD, SEM, TEM, and EDS. The heterostructures show a unique morphology with an anisotropic heterojunction due to the presence of polar/nonpolar surface facets of ZnO nanorods and different epitaxial relationship between CdO and the ZnO facets. A CdO cube with the growth direction of [111] was grown on the top polar surface of ZnO nanorod, while a homogenous CdO layer with the [010] direction is formed on nonpolar, {1010} side surface of ZnO nanorod. This anisotropic crystallographic heterojunction formed in a single nanostructure expectedly produces different electrical and optical properties, and should be considered for design and fabrication of heterostructure nanomaterials and nanodevices.

## Competing interest

The authors declare that they have no competing interests.

## Authors’ contributions

SHL and JX conceived the study and drafted the manuscript. SHL carried out the synthetic experiments and characterizations. CMP performed the TEM measurement and analysis. All authors read and approved the final manuscript.
